# Roma populations and health inequalities: a new perspective

**DOI:** 10.1108/IJHRH-01-2019-0004

**Published:** 2019-11-28

**Authors:** Lois Orton, Rachel Anderson de Cuevas, Kristefer Stojanovski, Juan F. Gamella, Margaret Greenfields, Daniel La Parra, Oana Marcu, Yaron Matras, Celia Donert, Diane Frost, Jude Robinson, Eve Rosenhaft, Sarah Salway, Sally Sheard, Elizabeth Such, David Taylor-Robinson, Margaret Whitehead

**Affiliations:** Department of Public Health and Policy, University of Liverpool, Liverpool, UK; School of Public Health, University of Michigan, Ann Arbor, Michigan, USA; Department of Social Anthropology, University of Granada, Granada, Spain; Department of Society and Health, Bucks New University, High Wycombe, UK; Department of Sociology, University of Alicante, Alicante, Spain; Department of Sociology, Catholic University of the Sacred Heart, Milan, Italy; Linguistics and English Language, University of Manchester, Manchester, UK; Department of Public Health and Policy, University of Liverpool, Liverpool, UK; School of Social and Political Sciences, University of Glasgow, Glasgow, UK; Department of Public Health and Policy, University of Liverpool, Liverpool, UK; Sociological Studies, University of Sheffield, Sheffield, UK; Department of Public Health and Policy, University of Liverpool, Liverpool, UK; School of Health and Related Research, University of Sheffield, Sheffield, UK; Department of Public Health and Policy, University of Liverpool, Liverpool, UK

**Keywords:** Europe, Policy, Research, Health inequalities, Roma

## Abstract

**Purpose:**

The purpose of this paper is to explore the emergence of “Roma health and wellbeing” as a focus of attention in European research and in policy and the possible detrimental consequences of action founded on a generic representation of “Roma health.”

**Design/methodology/approach:**

Based on discussions with and research conducted by scholars who work directly with Roma communities across European regions from a wide range of academic disciplines it suggests how future research might inform: a more nuanced understanding of the causes of poor health and wellbeing among diverse Roma populations and; actions that may have greater potential to improve the health and wellbeing among these populations.

**Findings:**

In summary, the authors promote three types of research: first critical analyses that unpick the implications of current and past representations of “Roma” and “Roma health.” Second, applied participatory research that meaningfully involves people from specific self-defined Roma populations to identify important issues for their health and wellbeing. Third, learning about processes that might impact on the health and wellbeing of Roma populations from research with other populations in similarly excluded situations.

**Originality/value:**

The authors provide a multidisciplinary perspective to inform research that does not perpetuate further alienation and prejudice, but promotes urgent action to redress the social and health injustices experienced by diverse Roma populations across Europe.

## Introduction

### The Roma in Europe

Although overall numbers are widely contested^1^ Roma are thought to form the largest minority ethnic group in the European region (WHO, 2017). Roma comprise many different populations, largely concentrated in Central and Eastern Europe and the Balkans (EC, 2014a). Despite common misconceptions, less than 20 percent of Roma are believed to live an itinerant lifestyle (including seasonal mobility across regional and national borders), with the majority seeking to settle or required to do so through coercive policy directives (Matras, 2015a; Donert, 2010). Roma have faced generations of discrimination and persecution and tend to experience higher levels of poverty and lower access to education and employment than majority populations (Sigona and Vermeersch, 2012; Tanner, 2005). Roma women can additionally face various forms of gender-based discrimination and violence across the lifecourse (including forced sterilisation) that compound the effects of ethnic- and class-based disadvantages (Zampas and Lamačková, 2011; Bollini *et al.*, 2009; European Union Agency for Fundamental Rights, 2013). Like many “excluded” or “minority” groups, Roma populations have been hit particularly hard by the 2008 economic recession that has affected much of the European region (Ghosh, 2011).

### The emergence of “Roma health and wellbeing” as a research and policy concern

A growing body of research compares the health of Roma and majority populations. These studies suggest that despite considerable heterogeneity of circumstances, most Roma populations appear to suffer poorer health and wellbeing relative to non-Roma, including higher rates of communicable and non-communicable diseases, poorer self-rated child and maternal health and higher mortality rates (Cook *et al.*, 2013). In Italy, for example, estimates suggest that the infant mortality rate for Roma is almost three times the rate of the wider population (EUMC, 2003). Health indicators for Roma are often worse than for other groups in similarly disadvantaged social positions (e.g. as measured by social class - La Parra *et al.*, 2016) with Roma women experiencing the worst health of all (European Union Agency for Fundamental Rights, 2013). This suggests that Roma and Roma women in particular, face adverse differential treatment and experiences that increase their exposure to risk factors for poor health beyond those that can be explained by poverty. In other words, the intersection of gender- and ethnicity-based minority social identities create increased disadvantage due to overlapping systems of oppression (European Union Agency for Fundamental Rights, 2012). This threatens their “Right to Health,” a key component of the International Covenant on Economic, Social and Cultural Rights (United Nations, 2000).

Before the end of communist rule in Europe, the situation of Roma populations was mainly the concern of the governments of Central and Eastern Europe (where most Roma reside). Apart from some debate on the situation of “Gypsies” in Western Europe (Simhandl, 2006), it was not until eastward expansion of the European Union (EU) that European institutions started to pay much attention to Roma peoples (McGarry, 2012). Today, the EU establishes the principles by which member states legislate on issues relating to Europe’s Roma populations. The health and wellbeing of Europe’s Roma has started to garner some political interest, with the production of a “Roma health report” in 2014 (EC, 2014a). Although no legally binding statements have been issued, improving the health and wellbeing of European Roma populations is an intended policy target of a growing number of strategies, most notably the 2005–2015 Decade of Roma Inclusion (Decade, 2005) and the 2011 EU Framework for National Roma Integration (EC, 2011). Policies adopt a largely country-specific approach, with little mention of extraterritorial considerations. Broadly speaking, the strategies aim to promote equal access to good quality housing, health care, employment and education for Roma populations. While reducing discrimination against Roma is a stated aim, very few specific actions are included (Fésüs *et al.*, 2012). Despite receiving increased attention, attempts to promote the health and wellbeing of Europe’s Roma populations have shown limited success. Reports suggest that differential morbidity and mortality rates between Roma and non-Roma continue to widen in most settings (Fésüs *et al.*, 2012; EC, 2014b).

### Multidisciplinary approaches to rethink the health and wellbeing of European Roma populations

The paper’s co-authors, researchers from the disciplines of history, linguistics, sociology, anthropology, public health, social epidemiology, social policy and law and from different European regions all of whom work with Roma populations, began a dialogue in the summer of 2016 to explore how diverse methodological approaches and analytical frameworks might be brought together to better understand and prompt effective policy action to improve the health and wellbeing of diverse Roma populations across Europe. This started with a workshop (convened by LO and RAdC) comprising a series of presentations and deliberative panel discussions (see Appendix for an overview of the workshop contributions) followed by the identification of research priorities. The propositions articulated at the workshop were then further explored and refined leading to the drafting of this manuscript. Members of Roma communities and non-governmental organisations from the UK, Spain, Italy, France, Slovenia, Bulgaria, Macedonia, Serbia, Kosovo, Hungary and Romania were active collaborators in the research presented during the workshop, including: design, data generation, analysis and interpretation; and have participated in the discussion and dissemination of selected workshop findings. Policy-makers and practitioners at local, regional, national and international levels, were engaged through co-authors’ networks, including: the World Health Organization, ministries of health, parliamentary groups, state councils and local health professionals, social workers and educators.

Below we outline how research might inform policy and practice to improve the health and wellbeing of European Roma populations. The limitations of existing data sets for understanding the health and wellbeing of specific Roma populations have been well rehearsed (see e.g. Kosa and Adany, 2007) and will not be repeated here. Instead we will provide practical suggestions – from our multidisciplinary perspective – for strengthening future research, policies and interventions. We recognise that research has great potential to do harm as well as good. If the wrong questions are asked, or valid questions addressed in the wrong manner, then the evidence produced could, and does, have very damaging implications for European Roma populations. In particular, there is danger that the act of exploring “Roma health and wellbeing” and singling Roma out for special attention perpetuates a paradigm in which Roma are seen as “different” or “deficient” when compared with majority populations (Dahinden, 2016; Matras, 2015b). Indeed, there is no denying that Roma have been treated differently in both research and in policy. Such distinctions can contribute to discrimination and separate treatment of Roma (Matras, 2015b). We argue that there is a strong case for interpreting the apparent “Roma disadvantage” as the result of the coalescence of multiple forms of disadvantage – particularly structurally rooted ethnic and gender discrimination and poverty and, in some cases, migration status – rather than a Roma-specific phenomenon. There must be careful consideration of the root causes that create the poorer health experienced by many Roma populations. We make some suggestions for how this might be achieved, below.

## Recommendations

### Pay careful attention when describing Roma populations

Little of the available evidence on the health outcomes and experiences of Roma populations carefully describes the characteristics of the population(s) concerned or the every-day contexts in which they live. Inappropriately generalised group characteristics are all too often implicated in the marginalisation of Roma and the singular “they” mentality that infuses much research and policy. This contributes to an overall notion of “Roma health” that overlooks the heterogeneity of experiences among diverse Roma populations (from the Gitano’s of Spain to the Romani of Kosovo). When coupled with an over-emphasis on individual- and community-level factors, such approaches reinforce cultural stereotypes and pathologise Roma as a “problem” while overlooking the intricate social, cultural and institutional factors that all too often create vulnerable circumstances for many Roma communities (Howard and Vadja, 2016). Future research should pay close attention to describing the varied characteristics of Roma populations, for example, whether the population is settled, travelling, migrant with a long-term settlement project, seasonally mobile, segregated or integrated, remotely located and living in urban or rural areas and sources of income and work practices. Descriptions of Roma populations should be complex and nuanced, based on self-identification whilst recognising that some Roma populations prefer to conceal their identity owing to fears of persecution (Ringold *et al.*, 2005). They should acknowledge the ever-changing nature of individual and group characteristics within Roma communities, the fluidity of such labels and their spatial and temporal specificity (Mir *et al.*, 2012).

### Apply theoretical frameworks and develop conceptual models

It is well accepted that health inequitalities are determined by complex multi-dimensional and interacting processes across the lifecourse from birth to old age (Graham, 2007); with the accumulation of either advantage or disadvantage across progressive life stages leading to the social distribution of health (Marmot and Wilkinson, 2005). We also know that many of these factors tend to cluster (and interact with one another) leaving some groups, such as Roma, with limited opportunity to evade underprivileged living conditions (Solar and Irwin, 2010). Future analyses should reveal the complexity of interacting processes at the governmental, societal, community and individual levels that compound one another to shape health and wellbeing (WHO, 2014). They should draw on theory and develop conceptual models to help make sense of complexity. Researchers could learn from case studies that successfully demonstrate how theory-driven approaches can be applied within other excluded populations, including indigenous populations; lesbian, gay, bisexual, trans and queer persons; or forced migrants. There is a multitude of theories that have proved fruitful for understanding the health of other populations in similar social positions. These include systems theory, intergenerational trauma and the minority stress model. Intersectionality theory (Crenshaw, 1991), for example, has emerged from feminist and critical race studies to show how interlocking systems of power related to class, race, gender and other social factors impact those who are most marginalised within society. Intersectionality theory has just started to be employed to bring closer attention to the multiple and overlapping nature of social factors that shape identities and underpin the discrimination, power imbalances and health experienced by European Roma populations (Kocze, 2009).

### Take a historical perspective and explore intergenerational effects

Most health inequitalities research focuses on a relatively limited time period. A historical perspective provides opportunities to explore long-term trends, as well as the impact of short-term phenomena. In Spain, for example, a longer-term view (from 1871 to 2005) reveals an overall positive trend for some health indicators (most notably infant mortality) among Roma populations. Further research is required to determine whether this represents a real change in the health disparities between Roma and non-Roma (Gamella and Martin, 2017). Historical approaches also provide opportunities to explore the legacy of past human rights abuses for present day Roma. Despite increasing recognition of the abuses brought upon Roma populations before, during and after the Holocaust (during which up to 50 percent of the European Roma population were exterminated; Stauber and Vago, 2007), an historical perspective exploring the legacy of these experiences for present day Roma peoples is largely absent in current research. Intergenerational approaches would help unpick the mental and physical health impact of trauma as it is transmitted across generations (Karpalski, 2006; Rosenhaft, 2006). As asserted by Powell and Lever (2017), we must study the long-term social processes that “shape asymmetric power relations between Roma and non-Roma” leading to their “outsider” status, ghettoization (or social and spatial marginalisation) and persistent persecution (p. 680). To date, much of the research with Roma populations is cross-sectional in nature and does little to monitor and evaluate whether health and social indicators have improved or worsened over time.

### Expose the impact of policy and practice on the experiences of Roma populations

More attention should be focussed on the social, political and economic structures and power differentials that shape the health-defining experiences of Roma populations. Future studies should analyse the way the health of Roma populations has been constructed and acted upon in the health system and in policy, and how this has been shaped by representations in research and the media (Greenfields *et al.*, 2015). Researchers should work directly with policy-makers, helping them to critically reflect on their role in shaping “Roma health” and exploring why key policies (such as the Decade of Roma Inclusion and the EU Framework for Roma Integration) have so far failed to make a difference to the health and wellbeing of European Roma populations. Sharing of best practices across different country settings could help identify promising future strategies. Research should also pay attention to the role that health professionals and interventions can play in alleviating or perpetuating Roma disadvantage through challenging or reinforcing traditional misconceptions, cultural stereotypes and stigma (European Union Agency for Fundamental Rights, 2013). Identification of good and bad practice could help direct actions that have the potential to narrow, rather than widen, the social and health inequalities between Roma and majority populations.

### Draw on the experiences of other stigmatised and marginalised groups

Opportunities exist to explore the potential for learning from successful strategies to improve the health of carefully selected groups living in broadly similar social, economic and environmental conditions. These might include, for example, the indigenous populations of Canada and Australia, Irish Traveller populations and lower caste groups in South Asia, among many others. In the Canadian setting, for example, a lack of “cultural continuity” (the integration of people within their culture and the transmission of traditional knowledge – i.e., the ability to “be who we are”) has been found to contribute to high suicide rates among indigenous youth (Chandler and Lalonde, 2008). Where possible, careful comparison of data specific to Roma populations with data relating to other identified population groups could inform an analysis of how different exclusionary processes impact on the health status of diverse Roma populations and help identify specific areas of focus to improve the health outcomes of Roma (and other) populations (see e.g. Powell and Lever, 2017).

### Embed meaningful participation of Roma populations

Research should challenge existing power structures and the legacy of social injustice by working with a variety of people from specific self-defined Roma populations as equal partners using participatory and action-oriented approaches to identify and address priority research issues (Marcu, 2015). Samples should be carefully selected, bearing in mind that many Roma prefer to conceal their identity for fear of continued persecution (Ringold *et al.*, 2005). Whilst challenging, when done well, such an approach could contest and challenge definitions and lines of action decided by researchers, practitioners and policy-maker, leading to more, dialogical, reflexive knowledge and courses of action. Indeed, any future research efforts that do not embed meaningful participation of Roma people would need a strong justification for this decision.

It should be noted that a participatory, intergenerational, intersectional approach designed to uncover the source of inequalities is likely to reveal issues within as well as outside Roma populations. Therefore, any engagement with marginalised and less powerful people within Roma populations will require careful negotiation. Any research will inevitably be influenced by social and cultural factors that determine access and willingness to participate. In order to maximise participation in the research process and control over the knowledge produced with specific categories of Roma (young people or people with low educational background, for example) it is necessary to choose accessible methods of engagement (artistic, visual performative) and support means of expression that are familiar to that particular target group (Marcu, 2015). Obstacles to participation may include literacy level or the unstable and precarious life conditions that many Roma populations find themselves existing in as well as social and cultural factors within their communities, which limit their possibility to take part in lengthy, academically formal research processes (Marcu, 2015). Future research should give priority to the problems perceived by Roma people, while involving all stakeholders in the process. A collaborative approach should be taken to reveal both told and untold invisible issues.

## Conclusion

This paper makes the case for research with Roma populations that digs deeper, embraces complexity, is more critical and political and develops trans-disciplinary theory-driven approaches to answer important and complex questions. Many of the challenges and research requirements we have laid out may equally apply to other minority ethnic groups (see Mir *et al.*, 2012, for example). What sets the Roma apart is the way in which the health experiences of distinct and heterogeneous populations in different settings and circumstances have been conflated and singled out in policy, the media, health care systems and in research. As a result, researchers need to exercise particular care not to perpetuate further alienation and prejudice, whilst at the same time seeking to promote urgent action to redress the social injustices experienced by diverse Roma populations across Europe. This is a difficult balancing act. The suggestions put forward here add some further considerations to this on-going debate.

## Appendix. Unpacking the multiple determinants of health for the Roma in Europe

### Workshop questions

1. What are the key processes – social, economic and political – that pose the main health threat for Roma communities?
  ■ How do these processes interact and compound one another over time?
  ■ How do they differ within and across different European contexts?
2. Can we map the key health gaps (between Roma communities and the general population) that result from these processes and how they differ across context and time?
3. In the light of international efforts to reduce health inequalities, are policies coherent in their diagnoses, explanations and interventions to improve the health of Roma communities?
4. What data are available and which methodological approaches might be most appropriate to investigate these issues?

Underpinning all of these questions is the over-arching goal of revealing the risks of ethnicising/racialising Roma by constructing the category of ‘Roma Health’ in research and policy and the implication that there is an inherent, causal link between Roma ethnicity and health inequality.

### Workshop presentations

Drs Lois Orton & Rachel Anderson de Cuevas (University of Liverpool, UK)

Overview of research pertaining to the health of European Roma populations.

Professor Yaron Matras (University of Manchester, UK)

Critical reflections on conceptualisations, definitions and problematisations of Roma in policy and research.

Dr Daniel La Parra (University of Alicante, Spain)

Roma and the World Health Organization conceptual framework on the social determinants of health.

Dr Celia Donert (University of Liverpool, UK)

Roma, human rights and population control in the Czech and Slovak Republics.

Dr Eve Rosenhaft (University of Liverpool, UK)

A historical perspective on medical and health discourses/issues in policies toward and the experience of European Roma.

Professor Dame Margaret Whitehead (University of Liverpool, UK)

Tracing social pathways to and from health inequalities – implications for developing more effective health and social policy.

Professor Juan Gamella (University of Granada, Spain)

Women's agency and reproductive health among the Romanian Roma.

Dr Oana Marcu (Catholic University of the Sacred Heart, Milan, Italy)

Irregular Romanian Roma migrants in Italy: the social context of drug use; and gender scripts, sexuality and health.

Professor Margaret Greenfields (Bucks New University, UK)

Roma migrants to the UK: housing and social work engagement as social determinants of health.

Kristefer Stojanovski (Center for Regional Policy Research and Cooperation, Studiorum, Macedonia)

The role of discrimination, policy, poverty and women's empowerment in determining the health of Roma populations.
